# Supplementary material for: Altered microRNA Transcriptome in Cultured Human Liver Cells upon Infection with Ebola Virus
Source: Int J Mol Sci. 2021 Apr 6;22(7):3792. doi: 10.3390/ijms22073792 (PMC8038836; doi:10.3390/ijms22073792)
Supplement: Supplementary file 1 [file ijms-22-03792-s001.zip › Supplementary_File/C_ GO_Analysis_Results/16-30nt_go_Reston-96h-Huh7_vs_Control-96h-Huh7_up.mature_mirna_targets/MF_result(Human).html]

| GO.ID | Term | Ontology | Count | Pop.Hits | List.Total | Pop.Total | Fold.Enrichment | Pvalue | FDR | Enrichment.Score | Gene.Ratio | GENES |
| --- | --- | --- | --- | --- | --- | --- | --- | --- | --- | --- | --- | --- |
| GO:0017017 | MAP kinase tyrosine/serine/threonine phosphatase activity | Molecular function | 2 | 13 | 103 | 17548 | 26.2106049290515 | 0.00255137287827906 | 0.940381724114093 | 2.5932260654416 | 0.0194174757281553 | DUSP18//DUSP9// |
| GO:0005231 | excitatory extracellular ligand-gated ion channel activity | Molecular function | 3 | 48 | 103 | 17548 | 10.6480582524272 | 0.00280432189746227 | 0.940381724114093 | 2.55217213682955 | 0.029126213592233 | GRIK5//GLRB//CHRNA1// |
| GO:0033549 | MAP kinase phosphatase activity | Molecular function | 2 | 14 | 103 | 17548 | 24.3384188626907 | 0.00296523432343547 | 0.940381724114093 | 2.52794098143835 | 0.0194174757281553 | DUSP18//DUSP9// |
| GO:0004708 | MAP kinase kinase activity | Molecular function | 2 | 15 | 103 | 17548 | 22.715857605178 | 0.00340836394425027 | 0.940381724114093 | 2.46745403757301 | 0.0194174757281553 | MAP2K7//MAPK3// |
| GO:0099095 | ligand-gated anion channel activity | Molecular function | 2 | 19 | 103 | 17548 | 17.9335717935616 | 0.00546658826821305 | 0.940381724114093 | 2.2622836350122 | 0.0194174757281553 | CFTR//GLRB// |
| GO:0008092 | cytoskeletal protein binding | Molecular function | 12 | 908 | 103 | 17548 | 2.25157178905949 | 0.00689275928181209 | 0.940381724114093 | 2.16160688825558 | 0.116504854368932 | PLEKHH2//WIPF2//SYN1//ABLIM2//MYOT//DCX//DYNC1I1//CLIP3//ARL4C//ARL8B//PLS1//AIF1L// |
| GO:0005031 | tumor necrosis factor-activated receptor activity | Molecular function | 2 | 24 | 103 | 17548 | 14.1974110032362 | 0.00865666535863148 | 0.940381724114093 | 2.06264937071291 | 0.0194174757281553 | TNFRSF11B//TNFRSF8// |
| GO:0005035 | death receptor activity | Molecular function | 2 | 24 | 103 | 17548 | 14.1974110032362 | 0.00865666535863148 | 0.940381724114093 | 2.06264937071291 | 0.0194174757281553 | TNFRSF11B//TNFRSF8// |
| GO:0017091 | AU-rich element binding | Molecular function | 2 | 25 | 103 | 17548 | 13.6295145631068 | 0.00937366241222867 | 0.940381724114093 | 2.02809069143308 | 0.0194174757281553 | ZFP36L1//DND1// |
| GO:0001078 | transcriptional repressor activity, RNA polymerase II proximal promoter sequence-specific DNA binding | Molecular function | 4 | 141 | 103 | 17548 | 4.83316119259106 | 0.00956331348670478 | 0.940381724114093 | 2.0193916077588 | 0.0388349514563107 | NFATC2//RORC//SKIL//NFE2L3// |
| GO:0005230 | extracellular ligand-gated ion channel activity | Molecular function | 3 | 75 | 103 | 17548 | 6.8147572815534 | 0.00976889153263113 | 0.940381724114093 | 2.01015471249133 | 0.029126213592233 | GRIK5//GLRB//CHRNA1// |
| GO:0003779 | actin binding | Molecular function | 7 | 410 | 103 | 17548 | 2.90873786407767 | 0.0105007599916838 | 0.940381724114093 | 1.97877926776337 | 0.0679611650485437 | WIPF2//PLS1//AIF1L//ABLIM2//PLEKHH2//SYN1//MYOT// |
| GO:0015276 | ligand-gated ion channel activity | Molecular function | 4 | 146 | 103 | 17548 | 4.66764197366671 | 0.0107690518458527 | 0.940381724114093 | 1.96782253219329 | 0.0388349514563107 | CHRNA1//GLRB//GRIK5//CFTR// |
| GO:0022834 | ligand-gated channel activity | Molecular function | 4 | 146 | 103 | 17548 | 4.66764197366671 | 0.0107690518458527 | 0.940381724114093 | 1.96782253219329 | 0.0388349514563107 | CHRNA1//GLRB//GRIK5//CFTR// |
| GO:0071889 | 14-3-3 protein binding | Molecular function | 2 | 27 | 103 | 17548 | 12.6199208917656 | 0.010884047732802 | 0.940381724114093 | 1.96320956225731 | 0.0194174757281553 | PI4KB//ZFP36L1// |
| GO:0043014 | alpha-tubulin binding | Molecular function | 2 | 31 | 103 | 17548 | 10.9915440025055 | 0.0142015532624011 | 1 | 1.84766415305367 | 0.0194174757281553 | ARL4C//ARL8B// |
| GO:0051015 | actin filament binding | Molecular function | 4 | 174 | 103 | 17548 | 3.91652717330655 | 0.0193297856939226 | 1 | 1.71377296089451 | 0.0388349514563107 | WIPF2//PLS1//AIF1L//ABLIM2// |
| GO:0099529 | neurotransmitter receptor activity involved in regulation of postsynaptic membrane potential | Molecular function | 2 | 38 | 103 | 17548 | 8.96678589678079 | 0.0209081975061448 | 1 | 1.67968340605737 | 0.0194174757281553 | CHRNA1//GRIK5// |
| GO:1904315 | transmitter-gated ion channel activity involved in regulation of postsynaptic membrane potential | Molecular function | 2 | 38 | 103 | 17548 | 8.96678589678079 | 0.0209081975061448 | 1 | 1.67968340605737 | 0.0194174757281553 | CHRNA1//GRIK5// |
| GO:0098960 | postsynaptic neurotransmitter receptor activity | Molecular function | 2 | 40 | 103 | 17548 | 8.51844660194175 | 0.0230234719408354 | 1 | 1.63782918413589 | 0.0194174757281553 | CHRNA1//GRIK5// |
| GO:0004712 | protein serine/threonine/tyrosine kinase activity | Molecular function | 2 | 41 | 103 | 17548 | 8.31067961165049 | 0.0241128575747673 | 1 | 1.61775131905374 | 0.0194174757281553 | MAPK3//MAP2K7// |
| GO:0016709 | oxidoreductase activity, acting on paired donors, with incorporation or reduction of molecular oxygen, NAD(P)H as one donor, and incorporation of one atom of oxygen | Molecular function | 2 | 44 | 103 | 17548 | 7.74404236540159 | 0.0275046671155574 | 1 | 1.56059360687678 | 0.0194174757281553 | FMO1//CYP26B1// |
| GO:0000981 | RNA polymerase II transcription factor activity, sequence-specific DNA binding | Molecular function | 12 | 1108 | 103 | 17548 | 1.84515088850724 | 0.0288840828871006 | 1 | 1.53934141740402 | 0.116504854368932 | DLX3//NFATC2//TFAP4//RORC//SKIL//NFE2L3//ZNF267//DMRT2//CREBL2//MIER3//ZFP36L1//TEAD3// |
| GO:0008138 | protein tyrosine/serine/threonine phosphatase activity | Molecular function | 2 | 46 | 103 | 17548 | 7.40734487125369 | 0.029866182716299 | 1 | 1.52482028229003 | 0.0194174757281553 | DUSP18//DUSP9// |
| GO:0000982 | transcription factor activity, RNA polymerase II proximal promoter sequence-specific DNA binding | Molecular function | 6 | 415 | 103 | 17548 | 2.46316528248918 | 0.0355057196707876 | 1 | 1.44970168014566 | 0.058252427184466 | DLX3//NFATC2//TFAP4//RORC//SKIL//NFE2L3// |
| GO:0008146 | sulfotransferase activity | Molecular function | 2 | 52 | 103 | 17548 | 6.55265123226288 | 0.0374087894236 | 1 | 1.4270263456686 | 0.0194174757281553 | GAL3ST1//HS3ST1// |
| GO:0042165 | neurotransmitter binding | Molecular function | 2 | 53 | 103 | 17548 | 6.42901630335226 | 0.038729970853911 | 1 | 1.41195282984042 | 0.0194174757281553 | GLRB//CHRNA1// |
| GO:0022838 | substrate-specific channel activity | Molecular function | 6 | 437 | 103 | 17548 | 2.33916153829064 | 0.0438280307186332 | 1 | 1.3582480426302 | 0.058252427184466 | CHRNA1//GLRB//GRIK5//KCNH8//CFTR//SLC14A1// |
| GO:0022824 | transmitter-gated ion channel activity | Molecular function | 2 | 57 | 103 | 17548 | 5.97785726452052 | 0.0441885607541149 | 1 | 1.35469014341904 | 0.0194174757281553 | GRIK5//CHRNA1// |
| GO:0022835 | transmitter-gated channel activity | Molecular function | 2 | 57 | 103 | 17548 | 5.97785726452052 | 0.0441885607541149 | 1 | 1.35469014341904 | 0.0194174757281553 | GRIK5//CHRNA1// |
| GO:0022839 | ion gated channel activity | Molecular function | 5 | 335 | 103 | 17548 | 2.54281988117664 | 0.0474420730230496 | 1 | 1.32383634235034 | 0.0485436893203883 | CHRNA1//GLRB//GRIK5//KCNH8//CFTR// |
| GO:0001227 | transcriptional repressor activity, RNA polymerase II transcription regulatory region sequence-specific DNA binding | Molecular function | 4 | 233 | 103 | 17548 | 2.92478853285554 | 0.0484366762984445 | 1 | 1.31482566558615 | 0.0388349514563107 | NFATC2//RORC//SKIL//NFE2L3// |
| GO:0022836 | gated channel activity | Molecular function | 5 | 338 | 103 | 17548 | 2.52025047394726 | 0.0489539968946288 | 1 | 1.31021184403616 | 0.0485436893203883 | CHRNA1//GLRB//GRIK5//KCNH8//CFTR// |
